# CaneFocus-Net: A Sugarcane Leaf Disease Detection Model Based on Adaptive Receptive Field and Multi-Scale Fusion

**DOI:** 10.3390/s25216628

**Published:** 2025-10-28

**Authors:** Xiang Yang, Zhuo Peng, Xiaolan Xie

**Affiliations:** 1 College of Computer Science and Engineering, Guilin University of Technology, Guilin 541006, China; 2 Guangxi Key Laboratory of Embedded Technology and Intelligent System, Guilin University of Technology, Guilin 541004, China

**Keywords:** sugarcane leaf diseases, CaneFocus-Net, CP, adaptive calibration mechanism, high-resolution shallow feature layer, nonlinear optimization strategy

## Abstract

In the context of global agricultural modernization, the early and accurate detection of sugarcane leaf diseases is critical for ensuring stable sugar production. However, existing deep learning models still face significant challenges in complex field environments, such as blurred lesion edges, scale variation, and limited generalization capability. To address these issues, this study constructs an efficient recognition model for sugarcane disease detection, named CaneFocus-Net, specifically designed for precise identification of sugarcane leaf diseases. Based on a single-stage detection architecture, the model introduces a lightweight cross-stage feature fusion module (CP) to optimize feature transfer efficiency. It also designs a module combining a channel-spatial adaptive calibration mechanism with multi-scale pooling aggregation to enhance the backbone network’s ability to extract multi-scale lesion features. Furthermore, by expanding the high-resolution shallow feature layer to enhance sensitivity toward small-sized targets and adopting a phased adaptive nonlinear optimization strategy, detection and localization accuracy along with convergence efficiency have been further improved. Test results on public datasets demonstrate that this method significantly enhances recognition performance for fuzzy lesions and multi-scale targets while maintaining high inference speed. Compared to the baseline model, precision, recall, and mean average precision (mAP50 and mAP50-95) improved by 1.9%, 4.6%, 1.5%, and 1.4%, respectively, demonstrating strong generalization capabilities and practical application potential. This provides reliable technical support for intelligent monitoring of sugarcane diseases in the field.

## 1. Introduction

As one of the world’s most important cash crops and primary sources of sugar, sugarcane plays a critical role in food processing, bioenergy production, and as an industrial raw material [1]. However, frequent occurrences of foliar diseases—such as mosaic disease, rust, and red rot—pose severe threats to the healthy growth of sugarcane [2], leading to sucrose degradation, yield losses, and substantial agricultural economic damage. Current monitoring methods primarily rely on expert visual assessment or conventional image processing techniques [3]. The former is inefficient and difficult to scale, while the latter is highly susceptible to variations in illumination, leaf overlap, and background interference in complex field environments, resulting in insufficient detection accuracy and robustness. In recent years, deep learning-based YOLO series algorithms have demonstrated significant potential in the field of agricultural pest and disease identification [4]. Nevertheless, existing models still face multiple challenges in sugarcane foliar disease detection. First, disease spots are often small and morphologically diverse, making small-object detection inherently difficult [5]. Second, in complex field scenarios, factors such as leaf occlusion, pose variation, and texture interference can distort feature extraction [6]. Furthermore, the trade-off between computational complexity and detection speed limits the deployment of existing algorithms on edge computing devices [7].

With the advancement of artificial intelligence, deep learning-based computer vision technologies have emerged as a research hotspot in agricultural disease detection [8]. Shahi et al. (2023) proposed two novel methods [9], the Measurement Index (MI) and Coefficient of Variation (CV), for estimating late leaf spot disease in peanuts using UAV-based remote sensing images. Their approach addresses the limitations of traditional methods by capturing the spatial distribution of disease symptoms within a plot. Similarly, a comprehensive review by Shahi et al. (2023) examined the progress in crop disease detection using UAVs and deep learning techniques [10]. They highlighted the importance of various sensors and image-processing techniques, categorized existing works, and discussed the performance of different machine learning and deep learning methods, emphasizing the challenges and opportunities in this field. Thite et al. (2024) introduced the “Sugarcane Leaf Dataset,” comprising 6748 high-resolution images classified into 11 categories [11], including nine diseases, healthy leaves, and dried leaves. This dataset serves as a valuable resource for developing machine learning algorithms for disease detection and classification in sugarcane. Furthermore, Daphal et al. (2023) focused on enhancing sugarcane disease classification using ensemble deep learning models [12]. Their study demonstrated the effectiveness of combining multiple deep learning models to improve classification accuracy, addressing challenges such as overfitting and model generalization. These studies collectively underscore the transformative impact of AI and deep learning in crop disease detection, providing insights and methodologies that inform the development of more accurate and efficient models.

In the field of sugarcane foliar disease detection, early studies primarily employed traditional image processing techniques. For instance, Ratnasari et al. (2014) proposed a detection method based on color space and threshold segmentation [13], combined with an SVM classifier, achieving relatively high accuracy. However, this approach suffered from large segmentation errors, heavy reliance on handcrafted features, and limited generalization capability. With the rapid development of convolutional neural networks (CNNs), Kumar et al. (2025) applied networks such as VGG-19 and EfficientNet for classifying sugarcane foliar diseases [14], with the EfficientNet model achieving a classification accuracy of 96.7% in experiments. Nonetheless, the model’s large parameter size, high training cost, and poor performance on small-scale lesion recognition posed significant limitations. To enhance the model’s capability in perceiving lesion location and morphology, Kumpala et al. (2022) incorporated YOLOv3 to develop a sugarcane red stripe disease recognition system [15], along with a web-based diagnostic tool. Trained on 4000 images via Google Colab, the system achieved a recognition accuracy of 95.90%; however, its simple image preprocessing pipeline, limited disease categories, and insufficient robustness restricted its applicability. To further improve model performance and generalization, Sun et al. (2023) introduced the SE-ViT model [16], which integrates a squeeze-and-excitation (SE) attention mechanism with a Vision Transformer, and incorporates a multi-head self-attention (MHSA) module alongside a support vector machine (SVM) for precise segmentation. This model achieved an accuracy of 89.57% on the SLD dataset, but its complex architecture, high computational cost, and limited generalization in complex environments remained challenges. To balance accuracy and efficiency, Li et al. proposed a hybrid model, SLViT [17], which fuses a lightweight CNN with a Vision Transformer. The model employs a ShuffleNetV2 backbone embedded with lightweight Transformer modules and enhances feature modeling capability through hybrid dilated convolutions and multi-head attention mechanisms (a summary of technical approaches is provided in Table 1). Although SLViT achieves a favorable trade-off between accuracy and inference speed, it lacks lesion localization capability and remains dependent on large-scale pretraining, and its adaptability for small-lesion detection and edge deployment still requires improvement.

Although these methods have achieved certain progress in specific scenarios, sugarcane foliar disease detection still faces significant challenges in small-object recognition, adaptation to complex backgrounds, lightweight deployment, and spatial localization. In this context, the primary objectives of this study are as follows: firstly, to enhance the model’s capability for detecting small-scale lesions in order to improve overall recognition accuracy; secondly, to design an efficient multi-scale semantic fusion architecture that strengthens feature extraction under complex background interference; in addition, to integrate a lightweight yet highly perceptive attention mechanism, thereby maintaining accuracy while controlling model parameter size; and finally, to optimize the trade-off between detection accuracy and inference speed, ensuring adaptability to application scenarios with varying computational resource constraints.

The remainder of this paper is structured as follows: Section 2, Materials and Methods, provides a detailed introduction to the dataset, the architecture of the CaneFocus-Net model, and its key technical components. Section 3, Results and Discussion, offers a comprehensive analysis of the experimental results and evaluates the performance and adaptability of each proposed enhancement across different scenarios. Section 4, Conclusion, summarizes the main findings of this study and discusses future research directions for sugarcane leaf disease detection.

## 2. Materials and Methods

### 2.1. Materials

This study employs a sugarcane leaf disease dataset provided by the Roboflow platform [18], comprising a total of 9100 images. Roboflow is a widely used public platform for computer vision dataset management, offering tools for data annotation, preprocessing, augmentation, and format conversion to facilitate the development and deployment of deep learning models. The dataset encompasses five common categories of sugarcane leaf conditions and covers multiple growth stages ranging from the seedling to the mature phase. It includes diverse lighting conditions, such as strong light, low light, and shadow, as well as typical background interference scenarios, including soil, weeds, and dew, thereby enhancing the model’s ability to recognize subtle features and complex disease patterns. Furthermore, prior to release, the dataset underwent Roboflow’s automated preprocessing pipeline, which involved image orientation correction, size standardization, contrast enhancement, adjustments to color saturation and brightness, and random rotation, thereby simulating a variety of real-world acquisition conditions. It is noteworthy that the dataset strictly adheres to academic research standards: all original images are copyrighted to the Roboflow Universe platform and are intended solely for model training and algorithm validation, without involving any field sampling or physical measurement processes.

**Figure 1:**
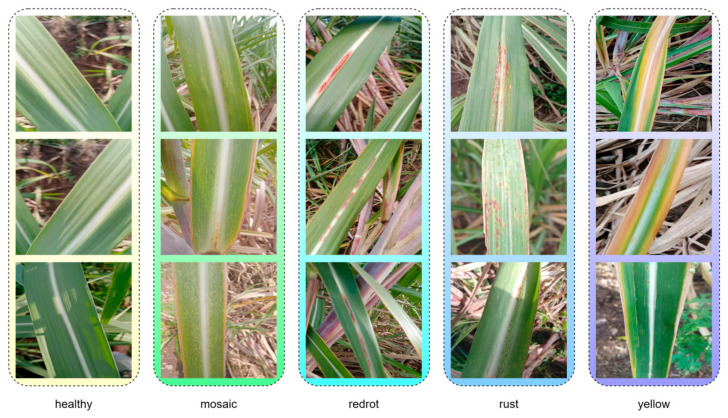
The five categories of the dataset.

The sugarcane leaf disease dataset used in this study comprises five categories: healthy, mosaic, redrot, rust, and yellow. Specifically, healthy refers to leaves that show no signs of disease and appear normal; mosaic indicates mottled patterns on the leaf surface, typically caused by viral infections; redrot refers to red rot disease, a fungal infection characterized by reddish-brown lesions on the leaves; rust denotes rust disease, which presents as rust-colored, powdery spore clusters on the leaf surface; and yellow represents leaf chlorosis, which may result from nutrient deficiencies or the early stages of disease. This classification system encompasses the major types of leaf diseases commonly observed during sugarcane cultivation and supports targeted automatic identification and diagnosis. Figure 1 illustrates representative image samples for each of the five categories, providing a visual reference for the dataset.

### 2.2. Establishment of a CaneFocus-Net Model for Identifying Sugarcane Leaf Diseases

#### 2.2.1. YOLOv11 Object Detection Network

The YOLO (You Only Look Once) series belongs to the family of one-stage object detection algorithms, which simultaneously predict the bounding boxes and classes of all objects in an image with a single forward pass, offering detection speeds far superior to two-stage algorithms. YOLOv1, structurally similar to GoogleNet [19], consists of 24 convolutional layers and 2 fully connected layers. With an input size of 448 × 448, the network produces a 7 × 7 × 30 feature matrix through its convolutional and fully connected layers. YOLOv2 improved detection accuracy while maintaining high speed and was capable of detecting up to 9000 categories [20]. YOLOv3 introduced a more powerful backbone network inspired by ResNet-101 [21], incorporated multi-scale feature fusion, and employed nine anchor boxes, significantly enhancing small-object detection performance. In 2020, Bochkovskiy et al. integrated multiple optimization strategies into YOLOv3 to develop YOLOv4 [22], which further improved accuracy and robustness. YOLOv5, released by Jocher et al. in 2020 [23], achieved outstanding performance on the MS COCO test-dev2017 benchmark, with YOLOv5x attaining 50.7% AP at an input resolution of 640. YOLOv7, published by the same author as YOLOv4 [24], surpassed all known detectors at the time in both speed and accuracy within the range of 5 FPS to 160 FPS. In 2023, Ultralytics introduced YOLOv8 [25], which replaced the anchor-based approach with an anchor-free design, allowing anchor determination to adapt automatically based on object center points rather than predefined priors. In 2024, Ultralytics released the latest iteration, YOLOv11 [26], which incorporated multiple structural optimizations over YOLOv8. These included replacing the C2f module with C3K2, introducing a C2PSA module with an attention mechanism after the SPPF block, adding two depthwise convolutions (DWConv) to the detection head, and adjusting model depth and width parameters. These modifications significantly reduced computational complexity and parameter count while maintaining detection performance. YOLOv11 is available in five variants—n, s, m, l, and x—with YOLOv11n offering the smallest model size and exceptional real-time performance. Given its superior performance over YOLOv5 and YOLOv8 in sugarcane leaf disease recognition tasks, YOLOv11n was selected as the baseline model for this study.

#### 2.2.2. CP Module

To enhance the model’s capability in detecting multi-scale sugarcane leaf lesions, this study fully replaces the C3K2 structures in the YOLOv11n backbone with CP modules that integrate the lightweight feature generation strategy of GhostNetV1 with the core concepts of GhostNetV2. The CP module, short for Cross Stage Partial Multi-Scale Feature Aggregation, is designed to enhance feature extraction by integrating information across different scales and network stages. It effectively improves the model’s ability to capture fine-grained details while maintaining computational efficiency through partial feature sharing and multi-scale fusion. GhostNetV1 employs Ghost modules to generate a portion of feature maps through computationally inexpensive operations [27], thereby reducing computational cost but inevitably weakening the model’s representational capacity. To address this limitation, GhostNetV2 introduces the DFC Attention mechanism [28], which strengthens fine-grained feature modeling and effectively improves detection accuracy in lesion regions. However, the attention mechanism in GhostNetV2 also increases architectural complexity and computational overhead. To further improve structural efficiency, the CP module incorporates the efficient Partial Convolution mechanism from FasterNet [29], which performs multi-scale modeling on only a subset of channels, thereby significantly enhancing computational efficiency. Finally, multi-scale features are fused through a 1 × 1 convolution, and residual connections are applied to reinforce feature propagation and improve network stability.

**Figure 2:**
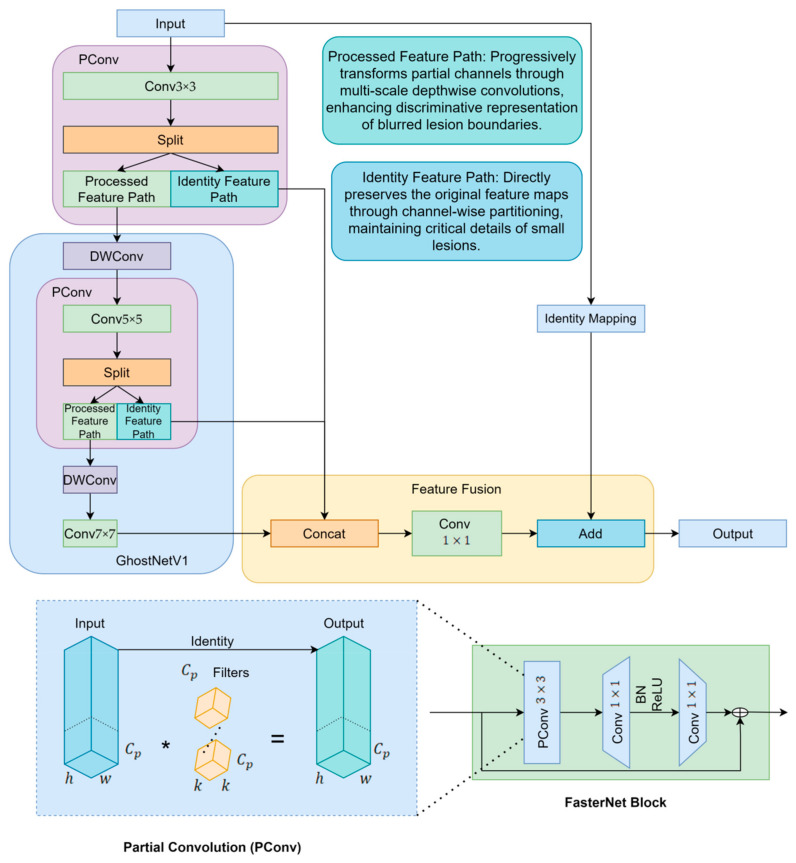
CP module structure diagram. The (*) symbol in PConv represents convolution operation.

The proposed CP module integrates the Cross Stage Partial (CSP) mechanism with a multi-scale feature aggregation strategy [30], and is composed of five structural units: channel split, multi-scale convolutional paths, identity mapping, feature fusion, and output interface. The input feature map is divided into two branches: one branch extracts multi-scale spatial features using convolutional kernels with different receptive fields (e.g., 3 × 3, 5 × 5, 7 × 7), thereby enhancing both local texture perception and global structural awareness; the other branch preserves the original features to form a residual connection, ensuring gradient stability. These two branches are concatenated along the channel dimension within the feature fusion module before being passed to the output. This architecture not only strengthens cross-scale information transfer between semantic layers (as illustrated in Figure 2) but also retains critical shallow-level detail features.

In the proposed CP module, the fusion ratio between the Ghost and PConv branches was not determined empirically but derived from the structural balance of feature flow within the module. The Ghost branch, inspired by GhostNetV1/V2, focuses on efficiently generating lightweight redundant features through inexpensive linear operations, which enhances representational diversity with minimal computational cost. Meanwhile, the PConv branch, following the partial convolution principle of FasterNet, processes a subset of channels to preserve fine-grained spatial and semantic information. To maintain a balanced information flow between efficiency-oriented and precision-oriented features, the CP module adopts an equal channel division (1:1 ratio) between the two branches. This ratio naturally emerges from the internal structure of the module, where feature maps from different sub-branches are concatenated along the channel dimension in equal proportions. Such symmetric feature organization ensures that each path contributes equivalent representational capacity to the final output, achieving structural equilibrium between lightweight expansion and detailed preservation. Therefore, the 1:1 ratio is not a manually tuned parameter but a design-driven result that reflects the inherent balance of the CP module’s feature aggregation mechanism. In sugarcane leaf disease detection, this structure significantly improves recognition accuracy for small lesions that are dense in number, irregular in shape, and variable in scale, while enhancing robustness under complex background conditions, all while maintaining controllable parameter size and high inference efficiency.

It is worth noting that the CP module is not designed to directly enhance discriminative power by itself but to optimize the efficiency and quality of feature representation within the backbone. Specifically, it improves the flow of spatial and semantic information through the joint use of Ghost-based lightweight expansion and PConv-based precise feature preservation, thereby providing a more balanced and expressive intermediate representation. However, lesion features in sugarcane leaves exhibit significant variations in scale and shape, which require enhanced receptive field adaptation and multi-scale aggregation for effective recognition. The SPPF-LSKA module complements the CP block by enlarging the receptive field and emphasizing key spatial responses through large-kernel attention, enabling the network to capture contextual dependencies and global feature patterns more effectively. Therefore, the observed improvement mainly reflects the synergistic effect between the structurally optimized CP module and the perceptually enhanced SPPF-LSKA module—the CP block refines and stabilizes the feature foundation, while SPPF-LSKA amplifies discriminative cues on top of these refined features. This synergy is indispensable because it integrates efficient feature generation with adaptive context modeling, leading to a substantial overall gain in detection performance.

#### 2.2.3. SPPF-LSKA Module

In this study, to further enhance the extraction of key features from lesion regions on sugarcane leaves, a channel-spatial adaptive calibration mechanism module—LSKA (Large Separable Kernel Attention)—is incorporated into the YOLOv11n backbone [31]. The LSKA module is derived from the Large Kernel Attention (LKA) design in the Visual Attention Network (VAN) [32]. By structurally reconfiguring the original LKA, the standard depthwise convolution and dilated depthwise convolution are replaced with two one-dimensional convolutions applied in the horizontal and vertical directions, forming a four-layer separable structure. This modification effectively expands the receptive field while substantially reducing computational cost and memory consumption. Compared with the original LKA rather than the baseline YOLOv11n structure, LSKA improves modeling capability and inference efficiency without introducing additional components, making it particularly well-suited for lightweight object detection tasks such as lesion detection. As a result, we acknowledge that, compared with the original YOLOv11n, the proposed SPPF-LSKA module does incur some additional computational cost due to the decomposition of the large 2-D kernel into two 1-D convolutions.

This improvement can be attributed to the structural decomposition of two-dimensional convolutions into a series or combination of one-dimensional convolutions along the horizontal and vertical directions, which effectively reduces the computational complexity from being proportional to the kernel area to being proportional to the kernel length. Specifically, a standard k × k convolution requires approximately k^2^ spatial multiplications and additions per position, whereas decomposing it into sequential k × 1 and 1 × k one-dimensional convolutions only requires about 2k operations. Therefore, the computational complexity is reduced from O(k^2^) to O(k), achieving roughly a k/2-fold reduction (for example, when k = 5, the computation is reduced by about 2.5 times). In the four-layer separable structure of the LSKA module, horizontal and vertical one-dimensional convolutions are alternately stacked and combined with dilation strategies, which not only maintain or even expand the receptive field (equivalent to using a larger or sparser 2D kernel) but also integrate spatial information in each layer at a lower computational cost, thereby further decreasing the total FLOPs.

The reduction in memory consumption mainly arises from two factors: first, the number of parameters is significantly reduced after decomposition (as the weight matrix decreases from k^2^ terms to two k-length terms), resulting in lower storage requirements; second, the size or number of intermediate activations is typically smaller, particularly when using depthwise or separable convolutions combined with pointwise convolutions for channel compression, which lowers the memory peak during forward and backward propagation. Finally, by stacking multiple small one-dimensional convolutions and introducing nonlinearities between layers, the four-layer separable design maintains or even enhances the model’s representational capacity—compensating for the expressiveness of a single large 2D kernel. Consequently, LSKA achieves an expanded receptive field and improved contextual modeling ability while substantially reducing computational cost and memory usage, making it more suitable for resource-constrained edge devices and real-time inference scenarios.

**Figure 3:**
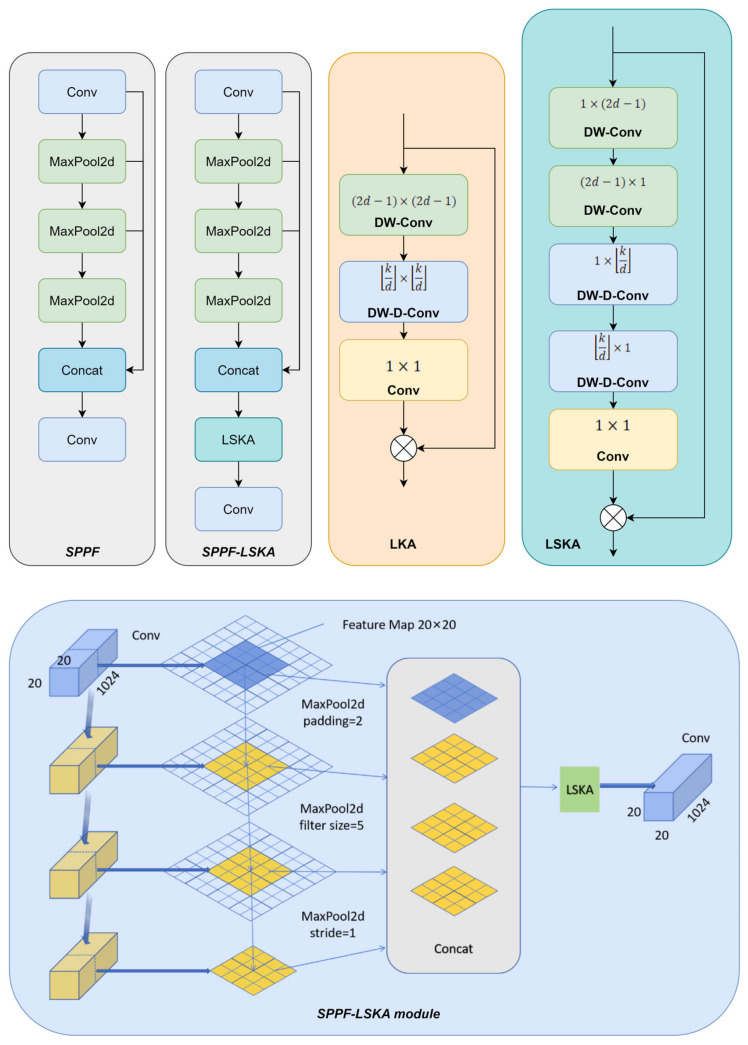
Comparison diagram of SPPF module improvement and different designs of large kernel attention module. Original LKA design in VAN including a standard depth-wise convolution (DW-Conv), a dilated depth-wise convolution (DW-D-Conv), and a 1 × 1 convolution. LSKA decomposed the first two layers of LKA into four layers, and each layer of LKA is formed by two 1D convolution layers. Notice that ⨂ represents Hadamard product, k represents the maximum receptive field, and d represents the dilation rate. The convolutional layers in this module adopt a kernel size of 5 × 5, stride of 1, and padding of 2.

In this study, the LSKA module was integrated into the SPPF component of YOLOv11n to construct an enhanced SPPF-LSKA module [33], thereby effectively improving the model’s ability to capture long-range dependencies and salient regions. The proposed module consists of two main components: (1) the original multi-scale max-pooling structure from SPPF—employing pooling kernels of different sizes (e.g., 5 × 5, 9 × 9, and 13 × 13)—to aggregate contextual features across varying receptive fields; and (2) the LSKA mechanism, which replaces conventional dot-product attention with a combination of directional one-dimensional convolutions (e.g., 1 × k and k × 1) or dilated convolutions. This approach approximates global attention in a more lightweight and localized manner, significantly reducing computational complexity and memory usage. Structurally, the input features are first processed through multi-scale pooling to generate semantically aggregated representations, which are then refined by the LSKA module to extract critical spatial and channel information. This guides the model to focus on lesion edges and fine-grained texture details, enhancing its sensitivity and localization accuracy for small lesion detection. The SPPF-LSKA module is designed to enhance multi-scale feature aggregation while emphasizing the most informative spatial regions through large-kernel attention. This mechanism shares conceptual similarities with recent advances in visual saliency modeling, such as the single-feature enhancement strategy proposed by Zhang et al. (2025) in “Video saliency prediction via single feature enhancement and temporal recurrence,” which strengthens discriminative features and highlights key visual cues [34]. By incorporating the LSKA mechanism, our module achieves a similar goal of emphasizing critical features to improve detection accuracy, particularly under complex visual conditions. The module achieves a balanced trade-off between accuracy and real-time performance, demonstrating its effectiveness and practicality in sugarcane leaf disease detection tasks. A comparative illustration of the improved module is shown in Figure 3.

#### 2.2.4. Small Object Detection Layer

**Figure 4:**
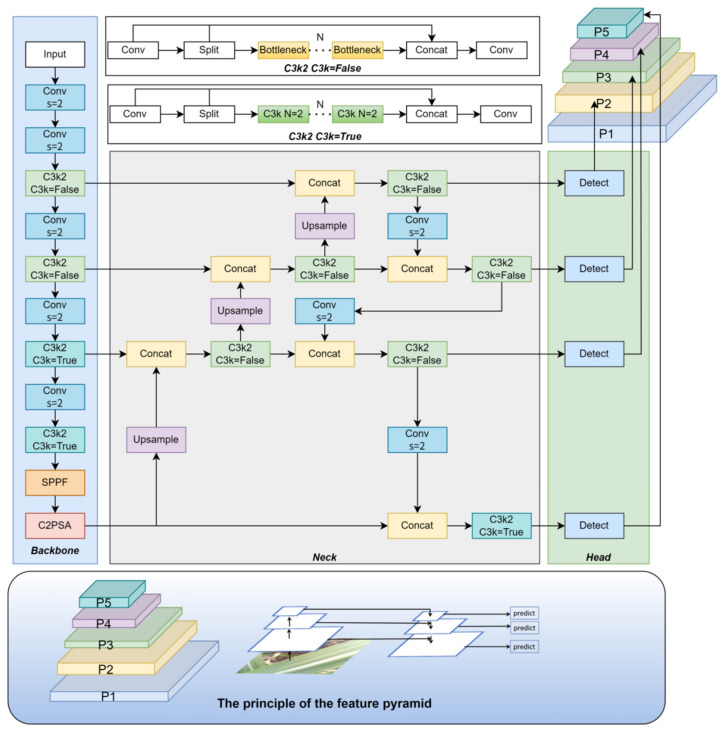
Structure diagram after adding small object detection layer.

To enhance the detection and localization capabilities of small lesions in sugarcane leaf diseases, this study incorporates a dedicated small-object detection head into the improved YOLOv11 model [35], combined with a Feature Pyramid Network (FPN) architecture [36]. This integration effectively fuses low-level spatial details with high-level semantic information, thereby strengthening multi-scale feature processing capabilities. The structural schematic of this design is illustrated in Figure 4. This approach significantly improves the model’s robustness and detection performance in complex backgrounds, providing strong support for the precise identification and classification of minute lesions.

The YOLOv11 model by default employs three multi-scale detection layers—P3 (80 × 80), P4 (40 × 40), and P5 (20 × 20)—corresponding to different object sizes. Among these, the P3 layer primarily targets small-object detection. However, for lesions that are smaller in size, exhibit blurred edges, or contain weak texture information, there remains a risk of missed or incorrect detections. To enhance the detection and localization capabilities for lesions, this study introduces a higher-resolution small-object detection layer, P2 (160 × 160), thereby extending the model’s multi-scale perceptual range and significantly improving lesion recognition performance in complex environments. Although the addition of the P2 layer increases computational load and parameter count, experimental results demonstrate a substantial improvement in lesion detection accuracy. Given the scale diversity of sugarcane leaf diseases, the original large-object detection layer P5 is retained. Comparative experiments indicate that removing P5 leads to a marked decrease in mAP50-95, underscoring its indispensable role in detecting large lesion areas. Consequently, the finalized CaneFocus-Net model augments the original three-layer structure with the P2 detection layer, achieving precise recognition of multi-scale lesions while effectively balancing performance and complexity.

In the proposed detection framework, four prediction heads (P2–P5) are employed to handle targets of different scales. Each detection layer corresponds to a specific search grid, where each grid cell serves as an anchor point to predict the object’s bounding box offsets and class confidence scores. The dense search grid in lower layers (e.g., P2 and P3) focuses on small objects, while the coarser grids in higher layers (e.g., P4 and P5) are responsible for larger targets. This hierarchical search strategy enables the model to achieve a balance between detection accuracy and computational efficiency.

#### 2.2.5. WIOU-CIoU-Inner_Iou Loss Function

In disease object detection, the accuracy of bounding box regression directly affects recognition performance. Traditional IoU-based loss functions include GIoU [37], DIoU [38], and CIoU [39]. The default loss function used by YOLOv11 is Complete IoU (CIoU), which effectively improves localization accuracy by comprehensively considering the overlap area between the predicted and ground truth boxes, the distance between their center points, and the aspect ratio difference. The loss function is formulated as follows:

(1) $$L_{CIoU}=1-IoU+\frac{\rho^{2}\left(b,b^{gt}\right)}{c^{2}}+\alpha v$$

Here, $\rho^{2}(\cdot )$ denotes the Euclidean distance between the center points of the predicted and ground-truth bounding boxes. b denotes the center of the predicted bounding box, while $b^{gt}$ represents the center of the corresponding ground-truth box. c is the diagonal length of the smallest enclosing box that covers both the predicted and ground-truth boxes. α is a trade-off factor used to balance the loss components, and v quantifies the difference in aspect ratio between the predicted box and the ground-truth box.

Although CIoU performs well in most general object detection tasks, it still exhibits certain limitations in fine-grained detection scenarios such as sugarcane leaf disease identification, which often involves small targets, blurred boundaries, and fine-grained textures. In particular, CIoU tends to underemphasize “hard samples” during the early training stages, making it prone to inaccurately fitting lesion regions with ambiguous boundaries or atypical appearances, thereby negatively affecting the overall detection accuracy.

To address this limitation, the proposed CaneFocus-Net model incorporates an enhanced loss function—WIOU-CIoU-inner_iou—which offers improved robustness and task adaptability. This loss function extends the original CIoU by integrating two key mechanisms: a WIOU-based weighting scheme [40], and an inner_iou structure-aware factor [41]. Specifically, WIOU introduces a dynamic weighting strategy based on the IoU value, enabling the model to adaptively focus on samples of varying regression difficulty. The corresponding formulation is as follows:

(2) ω=1−exp(−β·IoU)

Here, β denotes the attenuation control parameter. In the proposed Wise-IoU (WIoU) loss, the decay control factor *β* is not a fixed hyperparameter but a dynamically updated value during training. At the beginning of training, β is approximately equal to 1, since the average IoU of the first batch is initialized to 1. As the training progresses, the batch-wise average IoU is continuously updated, causing β to adaptively vary according to the distribution of IoU values. This dynamic mechanism allows the model to automatically emphasize harder or less accurate samples while reducing the loss contribution from well-fitted ones, thereby stabilizing convergence and improving overall localization performance. When the IoU is relatively low, ω takes a larger value, guiding the model to pay greater attention to hard samples with high regression errors, thereby enhancing overall regression robustness. Conversely, as the IoU approaches 1, ω tends toward zero, effectively suppressing overfitting on easily regressed samples. Incorporating this adaptive weighting mechanism, the formulation of WCIoU is defined as follows:

(3) $$L_{WCIoU}=\omega L_{CIoU}$$

In addition, to address the challenges posed by complex lesion morphology and irregular target regions, an inner_iou term is introduced. This term evaluates the intersection-over-union within the internal structural regions of the predicted and ground-truth boxes, explicitly modeling the spatial consistency and structural coverage of lesion areas. By doing so, it more effectively guides the model to fit the true shape of the target. The final overall loss function is formulated as follows:

(4) $$L_{WCIoU-CIoU-inner-iou}=\omega \cdot (L_{CIoU}+\lambda (1-{IoU}_{inner}))$$

Among them, λ is the balanced weight of the inner_iou term. The internal IoU weight coefficient λ is set to 1.3 by default to balance the contribution of the inner IoU term in the total loss, ensuring stable optimization and accurate box regression.

To more accurately evaluate the model’s localization ability for lesions with blurred edges, we construct an inner bounding box inside the original ground-truth box. The inner box keeps the original center and reduces the width and height by a scaling factor ratio. In this work, the default ratio is 0.7. The coordinates of the inner box are calculated as follows:

(5) $$w_{inner}=w\times ratio,h_{inner}=h\times ratio\;{x1}_{inner}=cx-w_{inner}/2,{x2}_{inner}=cx+w_{inner}/2\;{y1}_{inner}=cy-h_{inner}/2,{y2}_{inner}=cy+h_{inner}/2$$

It should be noted that an inappropriate scaling factor may exclude the core lesion area, thereby affecting localization accuracy. Specifically, when the inner box ratio is too small (e.g., 0.5), the inner box only covers the core of the lesion, and the blurred edges may be excluded, resulting in increased localization error; when the ratio is moderate (0.6–0.8), the inner box covers both the lesion core and the blurred edges, yielding minimal localization error; when the ratio is too large (e.g., 0.9), the inner box is close to the original bounding box, reducing the emphasis on the core region and being more affected by edge noise, which also increases the localization error. Therefore, the inner box ratio should be selected in a moderate range to balance coverage of the core and edge regions.

Building upon the multi-dimensional constraints of CIoU, this loss function introduces refined weight allocation and structure-aware strategies, conferring three key advantages: firstly, it focuses on hard-to-fit small-object samples, thereby enhancing training convergence and stability; secondly, by incorporating a structure-aware mechanism, it increases sensitivity to lesion boundaries and textures, improving recognition of irregularly shaped and blurred-edge targets; finally, it effectively suppresses overfitting to easy samples, optimizing overall detection performance.

#### 2.2.6. CaneFocus-Net Network Architecture

In this study, several key improvements were made to enhance the performance of the sugarcane leaf disease detection model, using YOLOv11n as the baseline framework:

- CP Module: The original C3k2 module in YOLOv11n was replaced with the CP module to improve the model’s capability in detecting multi-scale lesion regions.
- LSKA Module: The LSKA module was integrated into the SPPF module of YOLOv11n to form the SPPF-LSKA structure, thereby enhancing the extraction of critical features from sugarcane leaf lesions.
- Detection Head Adjustment: A small-object detection head was added, while the original large-object output from the backbone was retained. These adjustments optimized the network architecture, making it more suitable for detecting small and medium targets, and enhancing the model’s performance on small lesion regions.
- WIOU-CIoU-inner_iou Loss Function: The WIOU-CIoU-inner_iou loss function was introduced to replace the original CIoU loss in YOLOv11n, aiming to improve the accuracy of bounding box regression.

**Figure 5:**
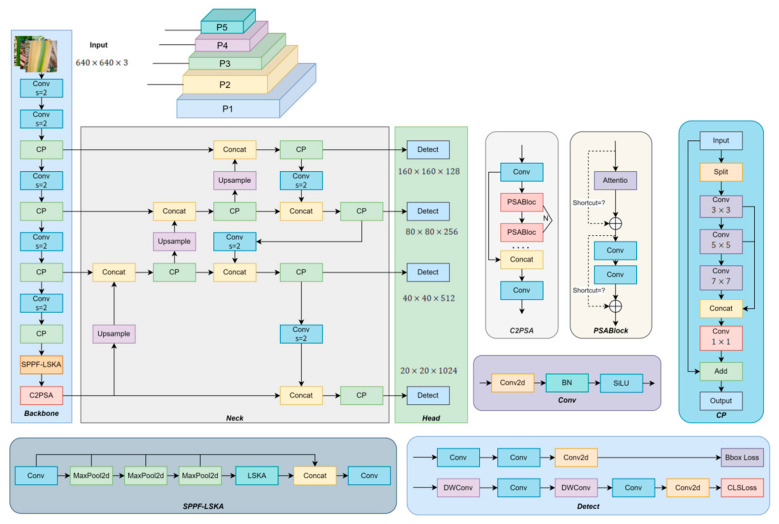
The structure of the CaneFocus-Net model.

As shown in Figure 5, the improved CaneFocus-Net model incorporates these enhancements, which collectively contribute to improved detection accuracy without significantly increasing the model’s complexity.

### 2.3. Experimental Environment and Parameter Settings

The experimental environment and main parameter settings used in this study are shown in Table 2.

### 2.4. Evaluation Index

To achieve accurate identification and detection of sugarcane leaf diseases, this study focuses on two key aspects: model accuracy and computational complexity. In this research, model size, the number of network parameters, Frames Per Second (FPS) and the number of floating-point operations required per forward pass, measured in GFLOPs (Giga Floating Point Operations) [42], is adopted as a key metric for assessing the model’s computational complexity. GFLOPs refers to the number of billions of floating-point operations required during a single inference, effectively reflecting the computational resource demand of the model in real-world deployment scenarios.

Meanwhile, recall (R), precision (P), and average precision (AP, %) are used to assess the detection accuracy of the model. AP, which combines both precision and recall, serves as a comprehensive metric for evaluating the overall performance of a model in object detection tasks. The mean Average Precision (mAP) is defined as the average of AP values across all object categories. The specific formulas for calculating these evaluation metrics are provided in Equations (6)–(9).

(6) $$P=\frac{TP}{TP+FP}\times 100\%$$

(7) $$R=\frac{TP}{TP+FN}\times 100\%$$

(8) $$AP=\int_{0}^{1}P\left(R\right)dR\times 100\%$$

(9) $$mAP=\frac{1}{n}\sum_{i=1}^{n}AP_{i}\times 100\%$$

Here, n denotes the number of classes in the dataset, i represents a specific class, TP stands for the number of true positives, FP denotes the number of false positives, and FN indicates the number of false negatives.

False positives are divided into two subtypes according to their causes: (1) background → lesion, and (2) lesion A → lesion B. Their mathematical definitions are given below.

(10) $${FP}_{bg->lesion}=\{p_{j}|\forall g_{i},\;IoU(g_{i}{,p}_{j})<\tau_{iou}\}$$

(11) $${FP}_{lesionA->lesionB}=\{p_{j}|\exists g_{i},\;IoU(g_{i}{,p}_{j})\geq \tau_{iou},\;c(p_{j})\neq c(g_{i})\}$$

Here, $p_{j}$ denotes the *j*th predicted box, $g_{i}$ represents the *i*th real box, $\tau_{iou}$ indicates IoU threshold (usually set at 0.5), $IoU(g_{i}{,p}_{j})$ denotes the intersection and union ratio between the predicted box and the real box, $c(p_{j})$ and $c(g_{i})$ represents category labels for predicted boxes and real boxes.

False negatives are further separated into two categories: (1) missed detections, and (2) detections suppressed by NMS.

(12) $${FN}_{missed}=\{p_{j}|\forall p_{k}\in p^{pre},\;IoU(g_{i}{,p}_{k})<\tau_{iou}\}$$

(13) $${FN}_{suppressed}=\{p_{j}|\exists p_{k},\;p_{l}\in p^{pre},\;IoU(g_{i}{,p}_{k})\geq \tau_{iou}\},\;IoU(p_{k},\;p_{l})>\tau_{nms},\;p_{l}\notin p$$

Here, $p^{pre}$ denotes all sets of prediction boxes before NMS, p denotes the final set of predicted boxes after NMS, $\tau_{nms}$ represents the NMS inhibition threshold, $p_{l}\notin p$ indicates the prediction box $p_{l}$ deleted by NMS.

In image object detection tasks, besides focusing on classification accuracy, the precision of bounding box regression must also be considered. Intersection over Union (IoU) is commonly used to measure the overlap between the predicted bounding box and the ground truth box, with a threshold set to determine positive samples (true positives, TP). Typically, an IoU threshold of 0.5 is used, meaning that a prediction with an IoU greater than 0.5 is regarded as a correct detection. The average precision calculated under this condition is referred to as mAP50. To further evaluate the model’s overall performance across different detection difficulties, the mAP50-95 metric is introduced. This metric averages the mean average precision (mAP) values computed at IoU thresholds ranging from 0.5 to 0.95 with a step size of 0.05, totaling ten thresholds. Compared to the single-threshold mAP50, mAP50-95 provides a more comprehensive reflection of the model’s robustness and generalization capability under varying precision requirements. In this study, the detection accuracy of sugarcane leaf diseases directly impacts the effectiveness of disease localization and classification. Therefore, both mAP50 and mAP50-95 are adopted as the primary evaluation metrics for detection accuracy to compare the performance of different models and analyze the most suitable improvements for sugarcane leaf disease recognition tasks.

To verify that the observed performance gains are not due to random fluctuations, we conducted paired bootstrap significance tests on class-wise AP values between competing models. Specifically, we performed 10,000 bootstrap resamplings to estimate the distribution of the mean AP differences and computed the corresponding two-tailed *p*-values. Statistical significance was marked using the conventional notation: *p* < 0.05 (*), *p* < 0.01 (**), and *p* < 0.001 (***). Results indicate that most performance improvements are statistically significant, confirming that the reported marginal gains are not random variations. To further validate the reliability of the reported improvements, we calculated paired bootstrap *p*-values for the key evaluation metrics. Specifically, P–P, P–R, and P–mAP denote the *p*-values corresponding to Precision, Recall, and mAP50, respectively.

## 3. Results and Discussion

### 3.1. A Comprehensive Comparison of Different Attention Mechanisms

To enhance the model’s perceptual capability for sugarcane leaf disease regions, this study incorporates multiple mainstream attention mechanism modules—including CAFM [43], CPCA [44], MLCA [45], SimAM [46], and LSKA—uniformly applied after the output of the C2PSA module within the YOLOv11 backbone. Their impact on model performance was systematically evaluated, with the corresponding experimental results presented in Table 3, providing a basis for selecting the optimal attention mechanism.

Table 3 shows that, compared to other improved attention mechanisms, the LSKA module achieves the highest mAP50-95. Although LSKA is not the top performer in Precision and Recall metrics, its superior mAP50-95 performance demonstrates a more robust and comprehensive enhancement in object detection tasks. Compared with other attention mechanisms such as SimAM, MLCA, CPCA, and CAFM, the Large Selective Kernel Attention (LSKA) module demonstrates superior capability in capturing long-range spatial dependencies while maintaining lightweight computation. Unlike SimAM, which focuses on pixel-level activation without explicit spatial adaptability, and MLCA or CPCA, which mainly emphasize multi-level or channel interactions, LSKA employs large kernel convolution and adaptive receptive field adjustment to enhance the extraction of global contextual features. This design effectively improves the model’s ability to focus on key regions across varying object scales. Therefore, integrating LSKA with the SPPF module (forming SPPF-LSKA) allows the model to combine multi-scale spatial feature aggregation from SPPF with the global attention refinement of LSKA, resulting in stronger feature representation, better localization precision, and improved detection performance for complex targets such as fine-grained disease spots on sugarcane leaves. Therefore, based on a comprehensive consideration of detection accuracy and generalization capability, this study ultimately selects the LSKA module for integration into the SPPF module to improve YOLOv11n’s recognition of complex lesion regions.

To further evaluate the robustness of the proposed model, a sensitivity analysis of mAP50 and mAP50-95 with respect to different combinations of the kernel size (k) and dilation factor (d) in the LSKA module was conducted. In our experiments, the LSKA was configured with a maximum kernel size of k = 41 and a dilation factor of d = 3, which were empirically found to provide an optimal balance between receptive field coverage and computational efficiency. It is worth noting that modifying k alters the maximum kernel size, which in turn affects the spatial dimensions of the input feature maps, causing the model to fail during training. Therefore, in this study, sensitivity analysis was performed only on the dilation factor d. The corresponding experimental results are summarized in Table 4, and the analysis demonstrates that moderate changes in d have a limited but observable influence on detection accuracy.

### 3.2. Ablation Experiment

To comprehensively evaluate the performance improvements contributed by each enhanced module in sugarcane leaf disease detection, this study constructs the improved CaneFocus-Net model based on YOLOv11 and designs ablation experiments to validate three key enhancements: integration of the lightweight structured attention mechanism LSKA into the backbone fused with the SPPF module, replacement of the original C3K2 structures with the CP module, and addition of the small-object detection head P2. The experiments adopt a controlled variable approach, independently or combinationally introducing these modules into the YOLOv11n architecture while maintaining consistent datasets, training epochs, and hyperparameters. This systematic analysis quantifies each module’s impact on precision and detection accuracy (mAP), with ablation results presented in Table 5.

As shown in Table 5, the model’s performance varied depending on whether individual modules or combinations of modules were introduced. The first group serves as the baseline using the original YOLOv11 architecture. In the second group, the lightweight attention mechanism LSKA was embedded into the SPPF module, forming the SPPF-LSKA structure. The third group replaced the original C3k2 module with the CP module, and the fourth group added only the small-object detection head P2. The results indicate that both the second and third groups improved the model’s precision and recall. Specifically, the second group achieved a higher mAP50, while the third group improved the mAP50-95. In contrast, adding only the P2 detection head, as in the fourth group, led to lower detection accuracy and precision compared to the baseline. This decline in performance is likely due to insufficient shallow semantic information and feature conflicts introduced by the additional detection head.

**Figure 6:**
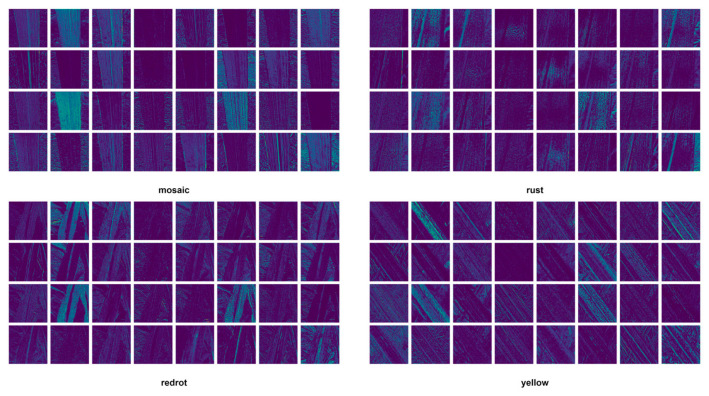
The visualization of P2 layer features for four categories.

To further examine the model’s performance in the early feature extraction stage, the P2 layer (corresponding to shallow feature output) was selected for visualization analysis. The P2 layer primarily captures low-level edge and texture information, and its activation patterns reveal how the model responds to different lesion types in the early processing phase. Figure 6 summarizes the comparative results of P2-layer feature responses for four representative sugarcane leaf diseases: yellow, mosaic, redrot, and rust.

From the figure, it can be observed that different disease types exhibit distinct shallow feature patterns. For yellow lesions, the model primarily focuses on linear venation and edge textures, but the overlap between lesion and background responses results in weak localization at this stage. The mosaic type shows complex, multi-directional texture activations, where the model struggles to separate lesion from non-lesion areas, indicating significant semantic confusion. For redrot lesions, the entire leaf region is highly activated, suggesting that shallow features rely excessively on color and brightness variations without meaningful semantic discrimination. In contrast, rust lesions display more localized and sparse activations that partially correspond to lesion regions, although boundary blur and noise interference remain issues. In summary, the P2-layer visualization results demonstrate that the model exhibits strong texture learning capability but weak semantic focusing at shallow levels. This finding highlights the necessity for deeper layers to enhance semantic representation of lesions through multi-scale feature fusion and attention mechanisms, thereby improving detection and localization performance.

The fifth group combined the SPPF-LSKA and CP modules. Compared to the second group (SPPF-LSKA only), this configuration improved recall, mAP50, and mAP50-95 by 2.4%, 0.6%, and 0.8%, respectively. Compared to the third group (CP module only), the proposed combination increased recall and mAP50 by 2.5% and 1.0%, respectively, while maintaining the same mAP50-95. These results demonstrate that the combination of the SPPF-LSKA and CP modules significantly enhances detection performance compared to using either module alone, with notable gains in recall, mAP50, and mAP50-95. The sixth group combined the SPPF-LSKA module with the P2 detection head, resulting in a 3.8% improvement in precision over the original YOLOv11n model. The seventh group combined the CP module with the P2 detection head. The eighth group incorporated all three improvements—SPPF-LSKA, CP module, and P2 detection head—and achieved the best overall performance. Compared to the baseline YOLOv11n model, this configuration improved precision, recall, mAP50, and mAP50-95 by 2.3%, 4.2%, 1.3%, and 1.2%, respectively. These results confirm that each individual module contributes to performance enhancement in different ways, and their combination produces a synergistic effect on detection precision and robustness. Although the proposed model incorporates multi-scale feature fusion, attention mechanisms, additional small-object detection layers, the detection performance for the “mosaic” and “yellow” categories remains relatively low. This is primarily due to the high visual similarity between these lesions and the background, as well as the limited number of training samples, which constrains the model’s discriminative ability for these categories.

To verify that the observed marginal gains are not due to random fluctuations, Table 6 presents the *p*-values corresponding to the evaluation metrics from the ablation experiments. These values indicate the statistical significance of the performance differences between the compared configurations.

The *p*-value results in Table 6 provide strong statistical evidence supporting the reliability of the observed improvements. Most *p*-values for Precision, Recall, and mAP_50_ are below 0.05, confirming that the performance gains introduced by the SPPF-LSKA, CP, and Head modules are statistically significant rather than random fluctuations. In particular, the consistently low *p*-values associated with Recall demonstrate that the SPPF-LSKA and CP modules effectively enhance the model’s feature representation and detection sensitivity. Furthermore, when all three modules are integrated, the overall *p*-values remain below the significance threshold, validating that the full configuration produces genuine, reproducible improvements rather than stochastic variations.

Table 7 compares the detection performance of YOLOv11n with and without the additional P2 layer under different NMS-IoU thresholds. The results show that introducing the P2 layer slightly increases recall but causes a notable drop in precision, especially when the NMS-IoU threshold rises from 0.7 to 0.9. This suggests that P2 generates more candidate boxes with high spatial overlap, leading to excessive suppression during NMS. Consequently, the added P2 layer introduces redundant detections rather than improving localization accuracy.

### 3.3. Improvement of Loss Function

**Figure 7:**
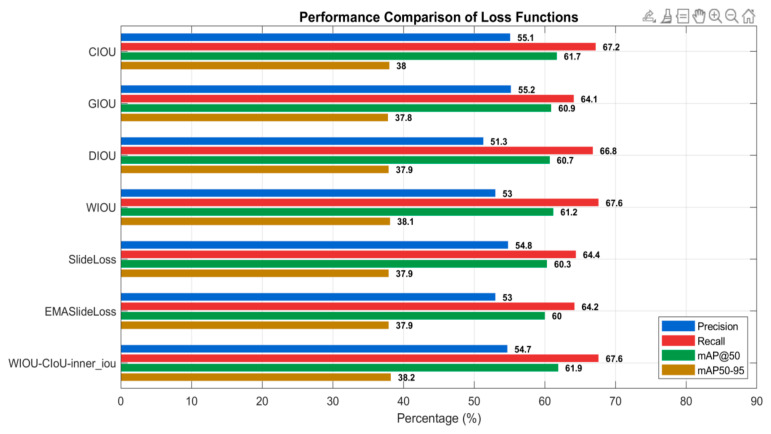
Experimental results of different loss functions.

Addressing the localization offset issues of YOLOv11 in lesion object detection, this study proposes an improved bounding box regression loss function—WIOU-CIoU-inner_iou—based on CIoU as the core. By incorporating weight adjustments and an inner IoU constraint, the proposed loss enhances the model’s ability to fit lesion edge structures more precisely. To validate its effectiveness, detection performance under the same improved model architecture (Group 8) was compared across multiple regression loss functions, including CIOU, GIOU, DIOU, WIOU, and the proposed WIOU-CIoU-inner_iou. Beyond the regression branch, exploratory optimizations were conducted on the classification branch by evaluating classification loss functions such as SlideLoss [47], and EMASlideLoss [48], assessing their robustness in complex backgrounds and small-object recognition scenarios. Detailed results are presented in Figure 7. The findings demonstrate that WIOU-CIoU-inner_iou outperforms existing methods across multiple metrics and significantly improves small-object detection accuracy, thereby validating its applicability and effectiveness in agricultural disease detection tasks. However, in the current work, we are unable to directly verify the specific improvements of this method on objects of different scales by plotting AP curves for each scale. This is mainly because the evaluation metrics in the YOLO series do not include AP_small, AP_medium, or AP_large, and converting results to compute these metrics may introduce accuracy loss. Therefore, using the available sub-metrics for overall performance evaluation is more reliable.

### 3.4. Comprehensive Comparison of Different Classical Models

To compare the detection performance of the improved model with commonly used classical models, the same dataset was used to train and validate established architectures such as Faster R-CNN [49], VGG-SSD [50], RT-DETR [51], YOLOv5s, YOLOv8n, and YOLOv11n. In this study, we report the inference speed of each model in terms of frames per second (FPS) to evaluate real-time performance. According to widely accepted standards for visual inference, an inference speed of 30 FPS is generally regarded as the threshold for real-time processing, while 60 FPS or higher ensures smooth frame-by-frame operation in high-frame-rate scenarios. This convention is consistent with prior empirical research. For example, Stepanenko and Yakimov (2019) demonstrated that optimized YOLO implementations can achieve real-time inference above 30 FPS on GPU-based systems [52], validating the practicality of this performance range. Therefore, the FPS results of our improved model confirm that its real-time performance is effectively maintained after the architectural modifications. The experimental results are presented in Table 8.

Table 8 indicates that Faster R-CNN and VGG-SSD exhibit relatively poor performance in sugarcane leaf disease detection, achieving mAP50 scores of only 30.2% and 33.1%, respectively. Moreover, these models possess large sizes and outdated architectures, making them inadequate for detecting small lesion targets and meeting deployment requirements. In contrast, the YOLO series demonstrates clear advantages in detection accuracy and model lightweight design. The mAP50 scores of YOLOv5s, YOLOv8n, and YOLOv11n progressively increase to 56.5%, 58.3%, and 60.4%, respectively, while model sizes become more compact. Building upon these results, the proposed CaneFocus-Net model achieves the best performance across all metrics, with a Precision of 54.7%, Recall of 67.6%, mAP50 of 61.9%, and an mAP50-95 improved to 38.2%. Although GFLOPs increase to 11.8 and the model size slightly expands to 6.21 MB, the overall resource overhead remains reasonable with a significant performance gain. Based on the theoretical FP16 peak performance of the RTX-3060 (approximately 25.3 TFLOPs), the theoretical simulated latency for YOLOv11n (6.3 GFLOPs) and CaneFocus-Net (11.8 GFLOPs) is approximately 0.25 ms/frame and 0.47 ms/frame, corresponding to theoretical FPS of around 4016 and 2146, respectively. It should be noted that these values are ideal estimates and do not account for memory access, CUDA core utilization, or batch size = 1 overhead; thus, the actual inference speed would be lower, but they can serve as a reference for FP16 simulated latency. Overall, CaneFocus-Net attains a favorable balance among detection accuracy, model compactness, and inference efficiency, making it a highly suitable detection model for sugarcane leaf disease recognition.

To further validate the generalization capability of the proposed model, additional experiments were conducted on two other publicly available datasets derived from the Sugarcane Computer Vision Dataset. These datasets contain diverse images of sugarcane leaves captured under different environmental and lighting conditions, providing a valuable basis for evaluating the model’s robustness and adaptability across various real-world scenarios. The experimental results are presented in Table 9.

As shown in Table 9, the proposed model maintains stable detection performance across different datasets, indicating its robustness and applicability in diverse real-world scenarios.

### 3.5. Image Recognition Results

**Figure 8:**
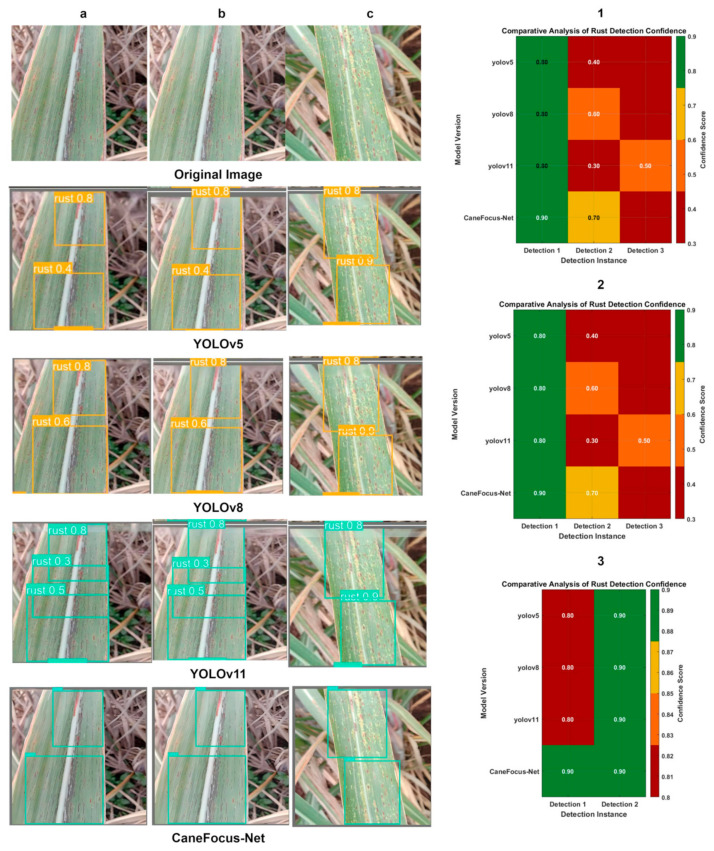
Comparison chart and corresponding heatmap of CaneFocus-Net model and YOLO series model test results. (**a**), (**b**) and (**c**), respectively, represent three different validation set images. The numerical values shown in the images indicate the confidence scores, which represent the probability that the model predicts the detected region as the corresponding disease class. (**1**), (**2**) and (**3**) are heatmaps corresponding to the comparison of several models for images (**a**), (**b**), and (**c**), respectively.

**Figure 9:**
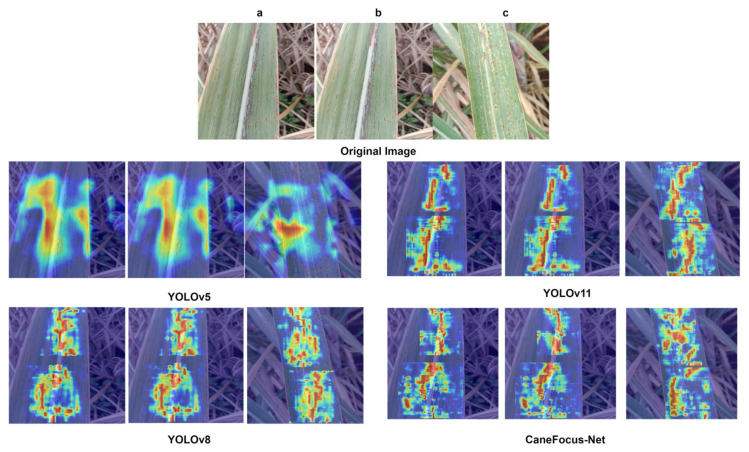
Comparison of experimental results of thermal maps with different models. (**a**), (**b**) and (**c**), respectively, represent three different validation set images.

This section focuses on analyzing the performance of the improved CaneFocus-Net model in recognizing sugarcane leaf disease images. The primary objective is to evaluate the model’s accuracy in identifying sugarcane leaf diseases, which is a critical task for optimizing sugarcane cultivation and enhancing agricultural efficiency. In the context of object detection, a confidence score is a numerical value assigned by the model to each predicted bounding box, representing the model’s certainty that the box contains an object of interest. Mathematically, it can be defined as:

(14) $$Confidence\;Score=P(Object)\times {IoU}_{pred,truth}$$

where P(Object) is the predicted probability that an object exists in the bounding box, and ${IoU}_{pred,truth}$ is the Intersection over Union between the predicted box and the ground-truth box. A higher confidence score indicates a higher likelihood that the predicted box correctly localizes an object. Figure 8 presents a comparative visualization of detection results on the sugarcane leaf disease dataset validation set between the CaneFocus-Net model and mainstream YOLO series models, Figure 9 supplemented by corresponding heatmaps that intuitively illustrate differences in detection performance among the models.

Figure 8 demonstrates that the CaneFocus-Net model exhibits a significant advantage in sugarcane leaf disease detection, particularly in typical lesion areas such as rust disease. The model’s bounding boxes precisely cover both the edges and central regions of the lesions, reflecting strong feature extraction and localization capabilities. Moreover, as illustrated in Figure 9, CaneFocus-Net visually suppresses false activations on background textures such as leaf veins, grass debris, and specular highlights, while concentrating its attention more effectively on true lesion regions. In contrast, models such as YOLOv5, YOLOv8, and YOLOv11 show issues including bounding box misalignment, incomplete coverage, or false detections, resulting in noticeably inferior recognition performance compared to CaneFocus-Net. This superiority provides an efficient and reliable technical foundation for intelligent recognition of sugarcane leaf diseases and automated agricultural disease diagnosis.

**Figure 10:**
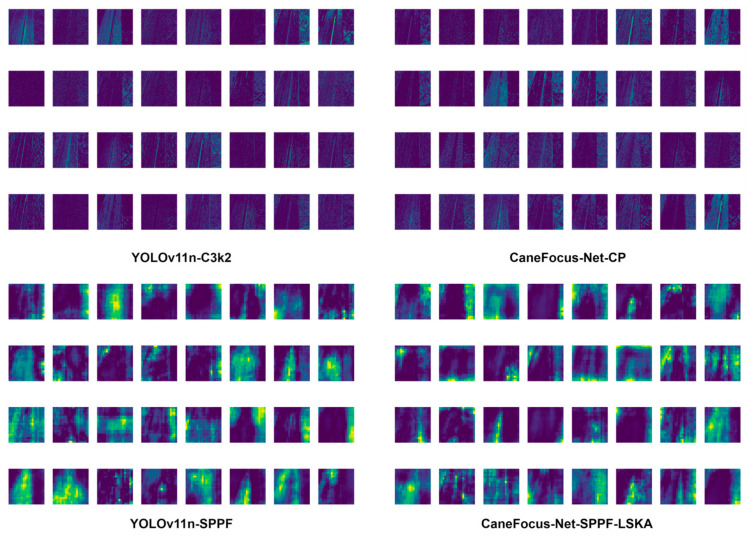
The intermediate feature maps of YOLOv11n and CaneFocus-Net.

Figure 10 presents the intermediate feature maps of YOLOv11n and CaneFocus-Net. In the baseline YOLOv11n (top), both shallow and mid-level layers exhibit strong activations on background textures such as veins and specular highlights, leading to feature noise. After integrating the CP and LSKA modules, the activation maps (bottom) become more focused on lesion regions, with background responses significantly weakened from the middle stage onward. This trend confirms that CP and LSKA progressively suppress redundant background activations and enhance discriminative lesion features, contributing to improved localization and robustness.

**Figure 11:**
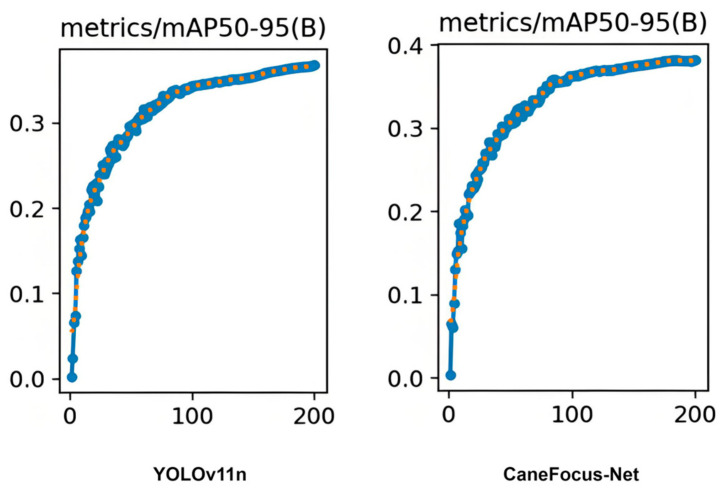
The mAP50-95 versus epoch for both YOLOv11n and CaneFocus-Net.

Figure 11 compares the mAP50-95 curves of YOLOv11n and CaneFocus-Net over the training epochs. As observed, CaneFocus-Net converges slightly faster and with smoother dynamics during the early training phase, suggesting improved training stability. However, both models reach similar plateau levels of approximately 0.36–0.38 mAP50-95, implying that the performance gain is primarily attributed to the architectural capacity introduced by CP and LSKA modules rather than optimization dynamics.

### 3.6. Discussion

**Figure 12:**
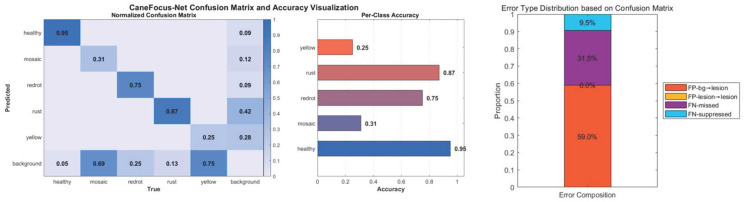
Normalized confusion matrix and visualization of category accuracy.

The experimental results demonstrate that the CaneFocus-Net model achieves mAP50 and mAP50-95 scores of 61.9% and 38.2%, respectively, significantly outperforming mainstream models such as YOLOv11n and YOLOv8n, while maintaining reasonable GFLOPs and model size. The incorporation of the P2 small-object detection head, multi-scale fusion modules, and attention mechanisms effectively enhances the model’s ability to detect small lesions on leaves, exhibiting particularly robust performance under complex backgrounds and varying natural lighting conditions. The normalized confusion matrix and class-wise accuracy of CaneFocus-Net are presented in Figure 12. The numbers of training and validation images for each category, along with the corresponding mAP values, are presented in Table 10.

## 4. Conclusions

The proposed CaneFocus-Net model demonstrates outstanding performance in sugarcane leaf disease detection, achieving significant improvements over traditional detection models in key metrics such as mAP50 and mAP50-95, thereby fully reflecting its superior detection capability. This model not only exhibits excellent detection accuracy and deployment potential but also holds promise for application in intelligent agricultural machinery and monitoring platforms to enable dynamic surveillance and precise control of sugarcane diseases. Furthermore, it can serve as a valuable reference for the detection of diseases in other crops, promoting the deep integration of precision agriculture, green agriculture, and sustainable agricultural development.

However, some limitations remain. Analysis of the normalized confusion matrix reveals that the “mosaic” and “yellow” classes exhibit relatively high confusion with the “background” class, which can lead to missed or incorrect detections in real-world applications. This confusion is primarily due to the visual similarity between these lesion types and the background leaf color, particularly under strong lighting conditions, leaf edge aging, or naturally textured leaf surfaces. Under such circumstances, the model struggles to accurately capture boundary differences, resulting in feature blending during the extraction phase—one of the key reasons for the suboptimal mAP performance. Additionally, as shown in Table 10, the model is undertrained on minority classes such as “yellow”, further exacerbating the confusion. The “yellow” class contains 1176 training and 83 validation images (approximately 16.3% of the dataset), indicating that it is not a severely underrepresented category. Despite this, its mAP50 and mAP50-95 remain the lowest (25.2% and 10.3%, respectively). Based on preliminary re-weighting trials, the potential gain for this class is very limited, suggesting that its low accuracy is mainly caused by inter-class visual similarity rather than data imbalance. Otherwise, simply due to insufficient training, the effect of the “redrot” would theoretically be worse. These findings suggest that despite the current architectural advancements, there is still room for improvement in the model’s fine-grained discrimination capabilities for different types of leaf lesions. However, it is worth noting that annotation consistency and dataset noise have a significant impact on the upper bound of detection performance. The ambiguity of lesion boundaries in the dataset may therefore partially limit the achievable mAP50 accuracy, preventing unrealistic expectations of further performance improvements.

Future research can focus on three key directions to further optimize the model. First, incorporating more refined attention mechanisms—such as saliency modules based on positional information and edge awareness—could guide the model to better focus on confusing regions. Second, enhancing the training of classes such as “mosaic” and “yellow” by employing techniques like contrastive learning, class reweighting, or hard sample mining could improve the model’s feature discriminability for these challenging categories. Third, improving the quality of data through more precise annotation of lesion boundaries and increased sample diversity would contribute to better generalization performance.

In addition, exploring multi-modal fusion, such as integrating texture, morphological, or hyperspectral information, could further support accurate lesion discrimination. This approach has the potential to fundamentally reduce feature overlap with the background, thereby improving the model’s accuracy and robustness in real-world applications.

## Figures and Tables

**Table 1:** The summarizing table of technical methods.

Author(s)	Year	Target Task	Methodology	Key Findings/Limitations
Ratnasari et al. [13]	2014	Early sugarcane leaf disease detection	Color space + Otsu threshold + SVM	Achieved basic detection, but suffered from large segmentation errors, hand-crafted features, poor generalization.
Kumar et al. [14]	2025	Sugarcane disease classification	VGG-19 and EfficientNet CNN models	EfficientNet reached 96.7% accuracy, but model was large, costly to train, and struggled with small lesion detection.
Kumpala et al. [15]	2022	Sugarcane red stripe detection + deployment	YOLOv3-based detection + Web-based diagnosis	Achieved 95.90% accuracy with 1.46 s response time; limited to single disease, simple preprocessing, low robustness.
Sun et al. [16]	2023	Enhanced accuracy via hybrid attention	SE module + MHSA (Transformer) + SVM segmentation	Accuracy 89.57% on SLD dataset; complex model, high computational cost, low adaptability to multiple diseases.
Li et al. [17]	2022	Lightweight classification with attention	ShuffleNetV2 backbone + lightweight ViT + HDC + MHSA	Good tradeoff between accuracy and efficiency; lacks localization ability; high pretraining demand.

**Table 2:** Experimental environment and Parameter Settings.

Configure	Parameter
CPU	AMD EPYC 7K62 48-Core Processor (AMD, Santa Clara, CA, USA)
GPU	NVIDIA GeForce RTX-3060 (NVIDIA, Santa Clara, CA, USA)
operating system	Windows10 64-bit system
accelerated environment	CUDA12.6
Pytorch	2.7.1
compiler language	Python3.10
momentum	0.937
initial learning rate	0.01
weight decay	0.0005
batch	32
epochs	200

**Table 3:** Experimental results of different attention modules. *p* < 0.001 (***).

Methods	Precision/%	P-P	Recall/%	P-R	mAP50/%	P-mAP	mAP50-95/%
YOLOv11n	52.8	0	63.0	0	60.4	0	36.8
YOLOv11n-CAFM	53.3	0.6383	60.1	***	59.1	0.0678	36.4
YOLOv11n-CPCA	52.5	0.7131	63.1	0.8932	59.8	0.6555	36.3
YOLOv11n-MLCA	53.8	0.2593	63.2	0.9368	60.0	0.1598	36.3
YOLOv11n-SimAM	49.1	***	63.6	0.4197	58.2	***	36.0
YOLOv11n-LSKA	51.9	0.3963	63.0	0.9776	59.7	0.3847	36.5

**Table 4:** Sensitivity Analysis of LSKA Hyperparameters.

Methods	Dilation Factor (d)	mAP50/%	mAP50-95/%
YOLOv11n-SPPF-LSKA	2	60.5	36.7
YOLOv11n-SPPF-LSKA	3	60.8	36.8
YOLOv11n-SPPF-LSKA	4	60.4	36.5

**Table 5:** Ablation experiment results. Bold represents the best group in this indicator.

ID	Model	SPPF-LSKA	CP	Head	P (%)	R (%)	mAP50 (%)	mAP50-95 (%)	Mosaic mAP50	Yellow mAP50
1	YOLOv11n	×	×	×	52.8	63.0	60.4	36.8	29.3	21.6
2	YOLOv11n	√	×	×	53.6	65.2	60.8	36.8	29.6	23.0
3	YOLOv11n	×	√	×	53.2	65.1	60.4	37.6	28.6	22.7
4	YOLOv11n	×	×	√	52.2	62.3	58.8	35.9	28.2	23.4
5	YOLOv11n	√	√	×	53.2	**67.6**	61.4	37.6	30.7	23.9
6	YOLOv11n	√	×	√	**56.6**	65.3	61.4	37.6	31.4	21.6
7	YOLOv11n	×	√	√	52.0	64.0	59.5	37.2	28.0	22.0
8	YOLOv11n	√	√	√	55.1	67.2	**61.7**	**38.0**	**32.1**	**24.5**

**Table 6:** The *p*-value results corresponding to the ablation experimental indicators. *p* < 0.05 (*), and *p* < 0.001 (***). Bold represents the best group in this indicator.

ID	Model	SPPF-LSKA	CP	Head	P (%)	P-P	R (%)	P-R	mAP50 (%)	P-mAP
1	YOLOv11n	×	×	×	52.8	0	63.0	0	60.4	0
2	YOLOv11n	√	×	×	53.6	0.1174	65.2	0.0232 (*)	60.8	0.2857
3	YOLOv11n	×	√	×	53.2	0.8632	65.1	0.0326 (*)	60.4	0.9608
4	YOLOv11n	×	×	√	52.2	0.6755	62.3	0.7387	58.8	0.1948
5	YOLOv11n	√	√	×	53.2	0.7908	**67.6**	***	61.4	0.0240 (*)
6	YOLOv11n	√	×	√	**56.6**	***	65.3	***	61.4	0.0612
7	YOLOv11n	×	√	√	52.0	0.4379	64.0	0.5123	59.5	0.3901
8	YOLOv11n	√	√	√	55.1	0.0432 (*)	67.2	***	**61.7**	***

**Table 7:** The NMS threshold ablation experiment.

NMS-IoU	Model	P2	P (%)	R (%)	mAP50 (%)	mAP50-95 (%)
0.7	YOLOv11n	×	52.8	63.0	60.4	36.8
0.8	YOLOv11n	×	51.1	59.4	57.8	35.8
0.9	YOLOv11n	×	44.8	49.8	51.2	32.9
0.7	YOLOv11n	√	52.2	62.3	58.8	35.9
0.8	YOLOv11n	√	49.9	58.1	56.3	35.1
0.9	YOLOv11n	√	40.8	51.8	50.2	32.5

**Table 8:** Experimental results of different typical models. *p* < 0.05 (*), and *p* < 0.001 (***). Bold represents the best group in this indicator.

Model	P/%	R/%	mAP50/%	P-mAP	mAP50-95/%	GFLOPs	Size/M	FPS
Faster R-CNN	30.2	26.8	30.2	***	19.5	134.51	315.16	18.90
VGG-SSD	33.1	37.0	33.1	***	15.1	15.29	104.04	32.57
YOLOv5s	52.6	63.3	56.5	0.0144 (*)	36.9	15.8	13.79	250
YOLOv8n	51.4	64.0	58.3	***	36.6	8.1	5.98	384.61
YOLOv11n	52.8	63.0	60.4	0	36.8	6.3	5.24	357.14
RT-DETR	53.5	61.9	58.6	0.3827	36.2	57.0	77.00	65.36
CaneFocus-Net	**54.7**	**67.6**	**61.9**	***	**38.2**	11.8	6.21	303.03

**Table 9:** Experimental results of different datasets.

Datasets	Model	P/%	R/%	mAP50/%	mAP50-95/%
sugarcane dataset v1	YOLOv11n	90.4	88.5	92.8	49.7
	CaneFocus-Net	92.0	87.0	93.5	49.5
sugarcane dataset v2	YOLOv11n	91.1	88.9	93	48.3
	CaneFocus-Net	91.3	90.4	92.9	49.1

**Table 10:** Experimental results of CaneFocus-Net for different categories.

Category	Training Images	Validation Images	mAP50/%	mAP50-95/%
healthy	2967	228	96.3	76.0
mosaic	1968	69	30.4	13.7
redrot	534	49	74.7	30.7
rust	1716	262	82.8	60.3
yellow	1176	83	25.2	10.3

## Data Availability

The data presented in this study are openly available at Roboflow. The website is: https://universe.roboflow.com/sugarcaneleaf/sugarcaneleaf-w0mto/dataset/2 (accessed on 23 July 2025).
